# Role of Exosomes in Brain Diseases

**DOI:** 10.3389/fncel.2021.743353

**Published:** 2021-09-13

**Authors:** Nan Zhang, Fengling He, Ting Li, Jinzhi Chen, Liping Jiang, Xin-Ping Ouyang, Lielian Zuo

**Affiliations:** ^1^Hengyang Key Laboratory of Neurodegeneration and Cognitive Impairment, Department of Physiology, Hengyang Medical School, Institute of Neuroscience Research, University of South China, Hengyang, China; ^2^Hunan Taihe Hospital, Changsha, China; ^3^Hunan Province Cooperative Innovation Center for Molecular Target New Drug Study, University of South China, Hengyang, China

**Keywords:** clinical treatment, biomarkers, cargo, brain diseases, exosomes, miRNAs

## Abstract

Exosomes are a subset of extracellular vesicles that act as messengers to facilitate communication between cells. Non-coding RNAs, proteins, lipids, and microRNAs are delivered by the exosomes to target molecules (such as proteins, mRNAs, or DNA) of host cells, thereby playing a key role in the maintenance of normal brain function. However, exosomes are also involved in the occurrence, prognosis, and clinical treatment of brain diseases, such as Alzheimer's disease, Parkinson's disease, stroke, and traumatic brain injury. In this review, we have summarized novel findings that elucidate the role of exosomes in the occurrence, prognosis, and treatment of brain diseases.

## Introduction

Extracellular vesicles are vesicles with a diameter range of 3 nm−1 μm secreted by cells into the extracellular space, which can be divided into exosomes (30–100 nm), microvesicles (100 nm−1 μm in diameter), and apoptotic bodies (50–5,000 nm) (Beeraka et al., 2020). According to the MISEV guidelines, extracellular vesicles measuring <100 nm in diameter, are termed as small extracellular vesicles (sEVs). Small extracellular vesicles (SEVs) originating from late endosomes are termed as exosomes, whereas other Small extracellular vesicles (SEVs) originate from the cell surface (plasma membrane) (Thery et al., 2018). Traditional methods of vesicle extraction and isolation are limited to in their ability to isolate different subtypes of EVs. Therefore, the terms “EV,” “sEV,” and “exosome” are used interchangeably in some studies (He et al., 2021b). This review focused on the function of exosomes.

Exosomes originate from the endomembrane system, and their envelope is continuously invaginated during early endosomal maturation to form intraluminal vesicles within the endosome. During this time, proteins, nucleic acids, and lipids are screened and enter the intraluminal vesicles. Late endosomes containing a large number of intraluminal vesicles are also called multivesicular bodies (Zhang et al., 2020; Nieland et al., 2021). Multivesicular bodies have two metabolic pathways. One is to be degraded by binding to lysosomes, and the other is to be transported to cell membranes, where the multivesicular membrane fuses with cell membranes and releases the inner vesicles into the extracellular space to form exosomes, which are loaded with proteins, non-coding RNAs, lipids and other biologically active substances (Ratajczak and Ratajczak, 2020). The endosome sorting complex is required for transport, with tetraspanins, ALG2-interacting protein X (Alix), heat shock protein (Hsp70), tumor susceptibility gene 101 protein, etc. being the accepted biomarkers for identifying exosomes (Budnik et al., 2016).

Exosomes carry various bioactive compounds as cargo, such as proteins, non-coding RNAs, lipids, etc. after being secreted from cells, thus facilitating communication between cells (Nieland et al., 2021). This function of exosomes forms the basis for their role in the development of various diseases, and altering the cargoes carried by exosomes or changing their surface molecules may hold therapeutic potential (Jafari et al., 2020). Exosomes are secreted by different types of cells and since the cargoes of exosomes secreted by the same type of cells differ in different disease processes suggests that studying the cargo of exosomes may be beneficial in predicting the course of a disease and for disease diagnosis (Zhang et al., 2020).

The brain is considerably intricate in its structure and function. The study of the molecular mechanisms underlying the development of brain disease is still in its infancy, creating limitations for clinical treatment. Brain diseases impose many social and economic burdens on society (Wang et al., 2020b). In recent years, exosomes have attracted considerable interest in the study of brain diseases, such as Alzheimer's disease, Parkinson's disease, stroke, and traumatic brain injury, due to their critical importance in the disease process and potential value for clinical application. The role and molecular mechanisms of exosomes carrying proteins related to the brain diseases [amyloid precursor protein (APP), α-synuclein (α-syn), mHtt, PrPsc] have been emphatically explored (Hartmann et al., 2017; Leblanc et al., 2017; Wang J. K. T. et al., 2017; Hill, 2019; Li B. et al., 2020; Pan et al., 2020; Perez-Gonzalez et al., 2020; Singh and Muqit, 2020; Tsunemi et al., 2020, 2021; Ananbeh et al., 2021; Soares Martins et al., 2021a). Notably, their ability to transport cargo is a key mechanism involved in the spread of disease. Compared to traditional therapeutic drugs, exosomes carrying drugs are more likely to pass through the blood-brain barrier (BBB), which helps the drugs to reach the target tissue (Azarmi et al., 2020). Due to the prevalence and easy availability of exosomes in the organism, as well as their involvement in various biomodulatory effects, exosomes have been considered as potential biomarker candidates for the clinical diagnosis and prognosis of diseases (He et al., 2021b). Over recent years, an increasing number of studies have explored the specific mechanisms of exosome involvement in brain disease (Soares Martins et al., 2021b). Here, we summarize novel findings that elucidate the role of exosomes in the occurrence, prognosis, and treatment of brain disease.

## Exosomes and Neural Tumors

Nervous system tumors include primary and metastatic tumors that originate in the brain, spinal cord, or meninges. As a highly malignant neural tumor, glioblastoma (GBM) has a high clinical mortality rate due to its poor prognosis, drug resistance, and susceptibility to hematologic metastasis. In recent years it has been closely studied in the field of exosome researches (De Leo et al., 2020; Ou et al., 2020).

Exosomes take part in the complicated inflammatory and immune responses of GBM. The inflammatory response present in GBM can alter the tumor microenvironment and promote tumor angiogenesis, cell proliferation, and invasive metastasis through a variety of active factors (Baig et al., 2020). Meanwhile, exosomes have been found to be involved in the inflammatory response in GBM and can alter the tumor microenvironment in GBM and promote tumor aggressiveness (Azambuja et al., 2020). Brain tumor-initiating cells transport tenascin-C through exosomes, which interacts with integrin α5β1 and αVβ6 to inhibit the mammalian target of rapamycin (mTOR) signaling pathway and further inhibit T cell activity (Mirzaei R. et al., 2018). LGALS9, a protein found in cerebrospinal fluid (CSF) exosomes derived from patients with GBM inhibits dendritic cell antigen presentation and cytotoxic T cell immunity (Yang et al., 2020). GBM cell-derived exosomes can promote the conversion of normal macrophages to tumor-associated macrophages (TAM), and TAM subsequently release large amounts of tumor growth-promoting exosomes. It has been further revealed that the inhibition of arginase-1+ TAM is a potential therapeutic target for GBM (Azambuja et al., 2020).

Exosomes and their cargo boost tumor proliferation and invasion in addition to altering the tumor microenvironment. Exosomes with cell adhesion molecule L1 (L1CAM) have been observed to stimulate the invasiveness and proliferation of GBM cells (Pace et al., 2019). The antisense transcript of hypoxia-inducible factor-1α is upregulated in exosomes of GBM cells, which can promote tumor viability, invasiveness, and radiation resistance (Dai et al., 2019). Polymerase I and transcript release factor (PTRF) in GBM cells accelerates the secretion of exosomes to transform the microenvironment and induces malignancy of adjacent cells. In both tumor tissue exosomes and blood exosomes isolated from GBM patients, tumor grade is positively correlated with the expression of PTRF in exosomes, and the expression of PTRF in blood exosomes decreases in patients after surgery (Huang K. et al., 2018).

Exosomal miR-301a derived from hypoxia-treated GBM cells can target TCEAL7 genes, thereby activating the Wnt/β-catenin signaling pathway and promoting the anti-radiation ability of the tumor (Yue et al., 2019). miR-182-5p is significantly upregulated in exosomes produced by GBM cells in a hypoxic environment, and this microRNA (miRNA) can inhibit the expression of Kruppel-like factors 2 and 4 (KLF2 and KLF4), leading to the accumulation of vascular endothelial growth factor (VEGF) receptor and promotion of tumor angiogenesis. Additionally, exosome-mediated miR-182-5p inhibits tight junction-related proteins (such as ZO-1, occludin, and claudin-5), thereby boosting vascular permeability and tumor transendothelial migration. Moreover, knockdown of miR-182-5p reduces angiogenesis and tumor proliferation (Li J. et al., 2020).

An abundance of abnormal nucleic acids in exosomes has been reported in GBM patients. A fragment of SOX2 DNA can be detected in exosomes, which is an important gene in embryonic stem cells (Vaidya and Sugaya, 2020). By measuring the serum exosomes in several patients with GBM, researchers found that the long non-coding RNA (lncRNA) HOTAIR 12q13 fragment, an RNA associated with GBM proliferation, is upregulated in exosomes, demonstrating that this RNA could be a new biomarker for GBM (Tan et al., 2018). However, these effects need to be explored further.

**Figure 1:**
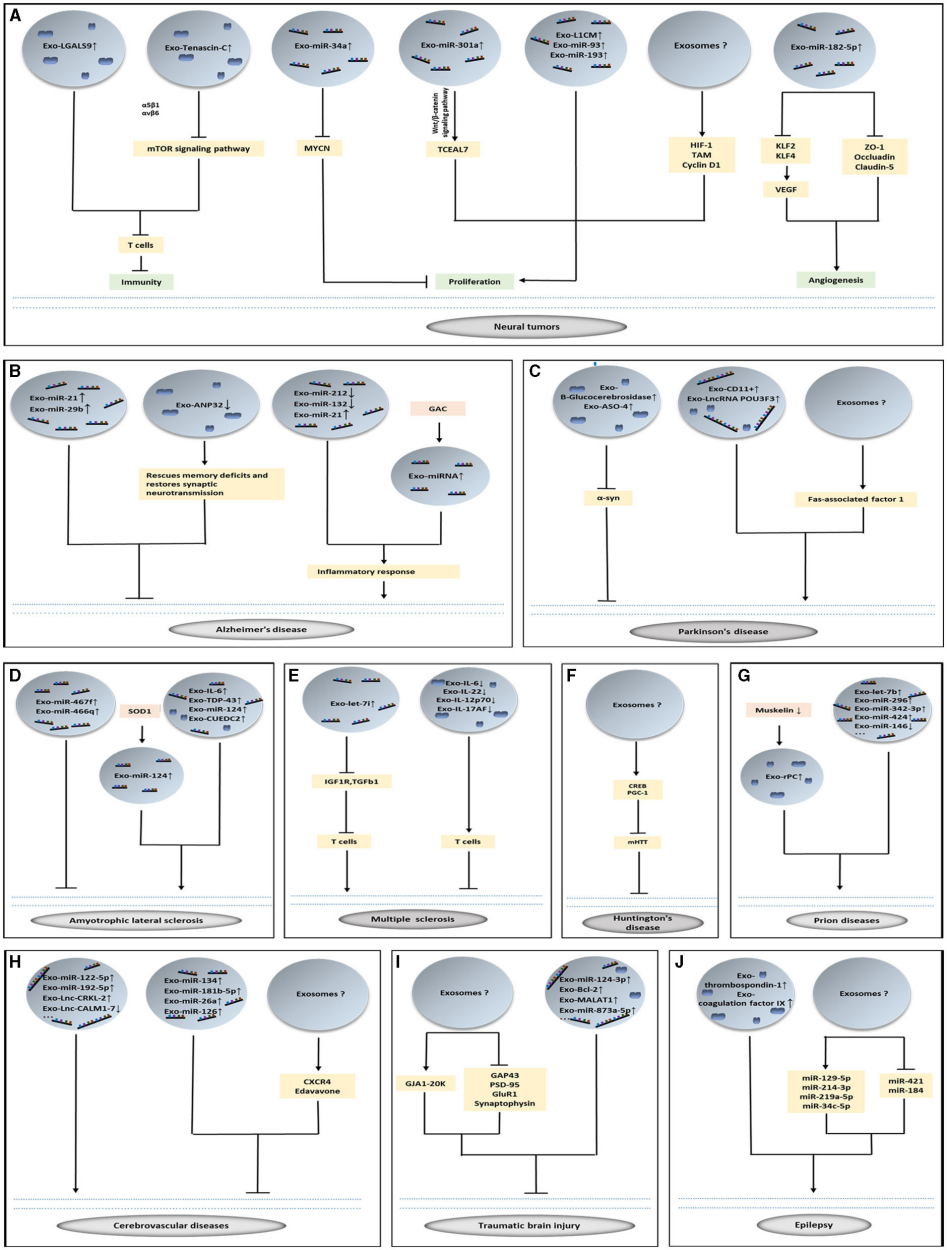
The role of exosomes in brain diseases. **(A–J)** Exosomes loading with vital cargoes promote or inhibit the occurrence of disease and play a therapeutic role in nervous system diseases.

Temozolomide (TMZ) is an oral capsule preparation for the treatment of GBM and overcoming resistance to this drug is of paramount importance. After treatment with TMZ, GBM cells produce exosomes containing miR-93 and miR-193 to target cyclinD1, which shortens the cell cycle and accelerates cell proliferation, thereby leading to drug resistance (Munoz et al., 2019). Exosomal miR-151a *in vitro* can improve the sensitivity of GBM cells to TMZ and have a therapeutic effect (Zeng et al., 2018). Recent studies have revealed that exosomes released from human bone marrow-derived mesenchymal stem cells (BMSCs) that are loaded with miR-34a alleviate the malignancy of tumors by silencing MYCN, thus promoting the sensitivity of GBM cells to TMZ (Wang et al., 2019a) (Figure 1A).

## Exosomes and Alzheimer's Disease

Among brain diseases, Alzheimer's disease is one of the most popular diseases studied in the field of exosome researches. The disparate modification of Amyloid beta (Aβ) peptide and tau protein in the damaged brain regions are considered characteristic features of Alzheimer's disease (AD). The former is degraded by APP (Luciunaite et al., 2020; Zhao et al., 2020). Over phosphorylation of tau proteins can lead to dissociation of tau proteins from microtubules and aggregation with each other, forming neurofibrillary tangles and deposition in neuronal cell bodies as well as axons and dendrites (Vandendriessche et al., 2020). The aggregation of abnormal proteins activates microglia and astrocytes, which in turn triggers a chronic inflammatory response, producing a variety of cytokines that can directly induce neuronal apoptosis and further Aβ accumulation in neurons. Therefore, neuroinflammation is an important factor in the development of AD disease (Soares Martins et al., 2021b).

Exosomes are involved in the complex mechanisms of secretion, spread, and degradation of Aβ or tau proteins. Researchers have investigated the physical properties of individual exosomes using electrostatic force microscopy, and observed that when higher concentrations of Aβ42 oligomers are fed to the neuroblastoma cells, the exosomes contained more Aβ42, implying that exosomes act as transport vesicles for Aβ42 (Choi et al., 2021). By constructing tau-containing N2a neurons, the researchers found that tau propagation between neuronal cells is facilitated by exosomes, and tau-containing exosomes are taken up by neurons and microglia, but not astrocytes. Additionally, on analyzing the CSF of patients with AD, it was discovered that the CSF exosomes contained tau in monomeric and oligomeric forms (Wang Y. et al., 2017). Among the exosomes generated from human induced pluripotent stem cell (iPSC)-derived neurons expressing mutant tau (mTau), there were a variety of unique proteins not found in normal exosomes, such as acidic nuclear phosphoprotein 32 family member A (ANP32A). In electrophysiological studies in human tau transgenic mice, knockdown of ANP32A rescued memory deficits and restored synaptic neurotransmission (Podvin et al., 2020).

Exosomes may play a role in the neuroinflammation observed in AD. Compared with healthy controls, patients with AD have higher levels of complement proteins in astrocyte-derived exosomes (ADEs), such as C1q, C46, and factor B. The mean levels of complement proteins in ADEs are significantly higher in the moderate dementia stage than in the preclinical stage. However, the complement regulatory proteins CD59, CD46, decay accelerating factor, and complement receptor type 1 (CR1) are lower in the ADEs of patients with AD than in healthy controls and decrease further with disease progression. This study suggests that measuring complement protein level in the exosomes may predict the progression of the disease (Goetzl et al., 2018). The exosomes produced by SHSWe cells contain miR-21 and can be internalized by microglia to promote an inflammatory response (Fernandes et al., 2018). AD mice demonstrate a high expression of glutaminase C (GAC) in their microglia, and previous studies have shown that GAC promotes exosome secretion and changes the exosome content to pro-inflammatory miRNAs, thereby activating the microglia (Gao et al., 2019).

Nucleic acids and proteins contained in the exosomes can be used as biomarkers for AD. miR-125b-5p, miR-451a, and miR-605-5p in CSF exosomes of patients with early dementia and elderly dementia are different from those in normal individuals (Mckeever et al., 2018). Additionally, miR-212 and miR-132 levels are decreased in the neural derived plasma exosomes from patients with AD (Cha et al., 2019). Synaptosomal-associated-protein-25 and the receptor for advanced glycation end products are expected to become the new biomarkers for AD (Agliardi et al., 2019). Additionally, some researchers are considering PIWI-interacting RNAs (piRNAs) as candidate biomarkers for AD (Jain et al., 2019). Growth associated protein 43 (GAP43), neurogranin, synaptotagmins, Rab3A, and synaptosome associated protein 25 in neuronal-derived exosomes are expected to serve as blood biomarkers for AD and mild cognitive impairment. Those proteins when used in combination can detect preclinical AD 5–7 years before the onset of cognitive impairment (Jia et al., 2021).

Many experiments have demonstrated that exosomes and the cargoes they carry can improve the symptoms of AD, but the specific molecular mechanisms still need to be investigated further (Soares Martins et al., 2021b). The expression of miR-21 was increased in exosomes produced by hypoxia pretreated mesenchymal stem cells (MSCs) suggesting that miR-21 can restore cognitive deficits in mice and prevent pathological features of AD (Cui et al., 2018). Delivery of MSC-derived EVs (including exosomes and microvesicles) to the brain *via* the intranasal route of administration (a non-invasive modality) can result in the inhibition of microglial activation and increase the density of dendritic spines (Losurdo et al., 2020). The exosomes produced by hippocampal neural stem cells protect the synapses in the hippocampus against the toxicity of Aβ oligomers and restore their long-term potentiation (LTP) and memory functions, which is a new method for the treatment of AD (Kim et al., 2020). Interestingly, exosomes produced by mature hippocampal neurons do not have this therapeutic function (Micci et al., 2019). Researchers have developed a neutral sphingomyelinase 2 inhibitor, called PDDC, which inhibits exosome release and is associated with the pathologic processes of exosomes (Sala et al., 2020). The oral administration of P2X purinoceptor 7 inhibitors in AD mice led to a significant improvement in the working and environmental memory, which may be due to inhibition of the release of microglial exosomes (Ruan et al., 2020). Injection of exosomes carrying miR-29b into the cornu ammonis 1 (CA1) region of the brains of AD mice resulted in reduced Aβ and improved performance in spatial learning and memory (Jahangard et al., 2020). Exosomes carrying quercetin demonstrate superior alleviation of the AD symptoms of mice than free quercetin by inhibiting cyclin-dependent kinase 5-mediated tau phosphorylation and reducing the formation of insoluble neurofibrillary tangles (Qi et al., 2020) (Figure 1B).

## Exosomes and Parkinson's Disease

The prominent pathological changes in Parkinson's disease (PD) are the degenerative death of dopaminergic neurons (DA) in the substantia nigra, a significant decrease in striatal DA content, and the appearance of Lewy bodies in the cytoplasm of residual nigrostriatal neurons (Singh and Muqit, 2020). α-synis a soluble protein expressed presynaptically and perinuclearly in the central nervous system and is associated with the pathogenesis and dysfunction of PD, and is a major component of Lewy bodies (Pinnell et al., 2021). α-syn is secreted in an exosome-dependent or non-exosome-dependent manner (Sun et al., 2020; Pinnell et al., 2021).

There is substantial evidence suggesting a strong link between exosomes and the development of PD. New research has revealed that exosomes can contribute to the intercellular spread of Fas-associated factor 1, which leads to the death of adjacent dopamine neurons, and is closely related to the disease progression of PD (Park et al., 2020). More direct evidence suggests that the presence of α-syn oligomers in CD11b+ exosomes produced by microglia/macrophages in the CSF of patients with PD induces α-syn aggregation within neurons (Guo et al., 2020).

Non-coding RNAs and proteins are abnormally expressed in the serum and CSF of patients with PD. Exosomal lnc-MKRN2-42:1 in the plasma has been positively correlated with the MDS-UPDRS III score in patients with PD (Wang et al., 2019b). lncRNA POU3F3 and α-syn in plasma L1CAM exosomes of patients with PD are increased, and this increase is related to a decrease in β-Glucocerebrosidase, as well as the disease severity of PD. The discovery of these three molecules may shed light on the mechanism of the autophagic-lysosomal system involved in PD pathogenesis (Zou et al., 2020). A new study shows that the exosomes released from neurons in the serum can be used to distinguish PD from atypical parkinsonism. Meanwhile, the concentration of α-syn in exosomes shows an increase with the disease progression of PD. This method of differential diagnosis can precede the clinical presentation (Jiang et al., 2020).

Some new findings demonstrate that exosomes may be beneficial in the treatment of PD (Sun et al., 2020; Yang et al., 2021). Some researchers have designed shRNA minicircles to treat PD (Li et al., 2020a). These RNAs are delivered by RVG-exosomes to act on the dopaminergic neurons and halt α-syn aggregation. Their data demonstrate that this kind of therapy is a long-term treatment for PD. Among the several PD treatments, the ideal treatment should be minimally invasive and effective in the long-term. Consequently, exosomal transport genes and the blocking of α-syn hold clear potential in this regard (Izco et al., 2019). Blood-derived exosomes from healthy volunteers attenuated dopaminergic neuronal damage in the substantia nigra and striatum of PD mice, resulting in improved motor coordination (Sun et al., 2020). Intracerebroventricular injection of exosomes loaded with antisense oligonucleotides (ASO)-4 into PD mice significantly ameliorated α-syn aggregation while attenuating the degeneration of dopaminergic neurons, resulting in significant improvements in motor function (Yang et al., 2021) (Figure 1C).

## Exosomes and Amyotrophic Lateral Sclerosis

Amyotrophic lateral sclerosis (ALS) is a disease that causes muscle weakness and atrophy of the limbs, trunk, and chest after a motor neuron injury. The pathogenesis of ALS includes an imbalance of protein homeostasis in the nervous system, prion-like proliferation and propagation of abnormal proteins, mitochondrial dysfunction, and an inflammatory cascade response. Mutations in the SOD1 gene lead to abnormal folding of superoxide dismutase 1 (SOD1) mutants *in vivo* and the eventual formation of toxic aggregates is responsible for the pathogenesis of ALS. TDP (TDR DNA-binding protein)-43 is the pathological marker protein of ALS, which causes re-entry of mature motor neurons into the cell cycle and induces apoptosis (Andjus et al., 2020).

Exosomes are engaged in the neuroinflammation observed in ALS. Interleukin (IL)-6 levels in plasma exosomes of astrocytes have been shown to be increased in patients with ALS, and IL-6 is positively correlated with disease progression within 12 months (Chen et al., 2019b). Motor neurons transfected with SOD1 can secrete exosomes containing inflammatory miR-124, and their co-culture with microglia *in vitro* can cause microglia to transform into the M1 type (Pinto et al., 2017). Exosomes produced by MSCs suppress the microglial pro-inflammatory phenotype in ALS mice *via* miR-467f and miR-466q (Giunti et al., 2021).

New studies have identified biomarkers in the exosomes of patients with ALS (Iguchi et al., 2016). TDP-43, a major component of ubiquitinated and hyper phosphorylated cytoplasmic aggregates observed in postmortem tissues of patients with ALS, is commonly found in the brains and is a major protein in the pathogenesis of ALS (De Boer et al., 2020; Suk and Rousseaux, 2020). The ratio of TAR DNA-binding protein-43 in the plasma exosomes demonstrated an increase with increasing follow-up time in patients with ALS (Chen P. C. et al., 2020). EVs, which contain exosomes, produced by the spinal cord tissue in ALS mouse models and ALS patients are rich in misfolded and non-native disulfide-cross-linked aggregates of SOD1, and the central nervous system-derived EVs in ALS mouse models are secreted by astrocytes and neurons, but not microglia (Silverman et al., 2019). The gene CUEDC2 in the CSF exosomes of patients serves as a biomarker for ALS (Otake et al., 2019). Proteomic analysis of CSF exosomes from ALS patients demonstrated a high level of novel INHAT repressor (NIR), while NIR is reduced in the nucleus of motor neurons (Hayashi et al., 2019) (Figure 1D).

Exosomes also play a role in the treatment of ALS. Exosomes derived from adipose-derived stem cells (ADSCs) could recover coupling efficiency, complex I activity, and mitochondrial membrane potential in an *in vitro* experiment related to ALS (Calabria et al., 2019). Repeated administration of ADSC-derived exosomes by intravenous and intranasal administration to ALS mice improved their motor performance, protected the lumbar motor neurons and neuromuscular junctions, and reduced microglial activation (Bonafede et al., 2020).

## Exosomes and Multiple Sclerosis

Multiple sclerosis (MS) is a type of autoimmune demyelinating disease caused by the loss of tolerance to a self-protein (myelin antigen) (Jackle et al., 2020). Its main pathological manifestations are the disruption of BBB integrity and infiltration of peripheral immune cells into the central nervous system to form inflammatory lesions, which in turn initiate autoimmune mechanisms leading to myelin destruction and axonal damage, as well as motor, sensory, and autonomic dysfunction (Martinez and Peplow, 2020).

Proteins and nucleic acids are connected with the pathogenesis of MS. Let-7i in circulating exosomes inhibits insulin like growth factor 1 receptor (IGF1R) and transforming growth factor beta receptor 1, thus inhibiting the differentiation of regulatory T cells and promoting the development of MS (Kimura et al., 2018). Exosomes in the CSF of patients with MS may contain high levels of ceramide and acid phosphatase, which are associated with axonal neurological dysfunction (Pieragostino et al., 2018). Myelin basic protein, proteolipid protein, and myelin oligodendrocyte glycoprotein are expressed in the serum exosomes of patients. Accordingly, exosomes may enhance and/or maintain the antimyelin immune response in MS (Galazka et al., 2018). Several researchers have summarized the miRNAs that are abnormally up or down regulated in the exosomes present in the CSF or blood of patients with MS, and have indicated that further studies will investigate their usefulness as biomarkers for determining the prognosis and therapeutic effects of MS.

Recently, researchers have also explored the role of exosomes in the treatment of MS. Exosomes secreted by human MSCs [stimulated by interferon gamma (IFN-γ)] can alleviate demyelination in MS mice, decrease the levels of proinflammatory Th1 and Th17 cytokines (including IL-6, IL-12p70, IL-17AF, and IL-22), increase the levels of immunosuppressive cytokines, and upregulate CD4+CD25+FOXP3+ regulatory T cells in the spinal cord of MS mice. These findings make cell-free therapy for MS a distinct possibility (Riazifar et al., 2019). It has been shown that exosomes produced by human umbilical cord blood-derived MSCs can inhibit the proliferation of peripheral mononuclear blood cells (PBMCs) when co-cultured with PBMCs *in vitro* (Baharlooi et al., 2021) (Figure 1E).

## Exosomes and Huntington's Disease

Huntington's disease (HD) is caused by a mis-expression of multiple CAG repeats (thus leading to Htt protein variation) on the *HTT* gene (He et al., 2021a).

The delivery of pathological proteins and miRNAs in different species is carried out through exosomes (a non-cell form), and these proteins and miRNAs trigger or inhibit HD-related behavior and pathology (Jeon et al., 2016). When fibroblasts from patients with HD were injected into the ventricles of newborn mice and induced pluripotent stem cells carrying CAG repeat sequences, researchers found the specific exosomal mHtt derived from the fibroblasts of patients with HD in these mice (Didiot et al., 2016). Meanwhile, mouse embryonic fibroblasts (MEFs) overexpressing exon 1 of the *HTT* gene showed that mHTT was found to be present in glutaminase 2-mediated exosomes when bound to exosomal structural proteins Alix and TSG101(Beatriz et al., 2021). Exosomal miRNAs have been found in HD, such as miR-22, miR-214, miR-150, miR-146a, and miR-125b (Wang J. K. T. et al., 2017; Reed et al., 2018). However, the mechanism of these miRNAs needs to be further investigated.

Exosomes can cross the blood-brain barrier and affect the nervous system to regulate mHtt aggregation, mitochondrial dysfunction, cell death and cell viability in HD (Lee et al., 2021). Exosomes secreted by ADSCs are considered critical for relieving HD phenotypes, which up-regulate phosphorylated CREB, PGC-1, and expedite non-apoptotic protein levels (Lee et al., 2016), notably alleviating mHtt aggregation in R6/2 mouse neurons (Deng et al., 2019; Lee et al., 2021). Thus, exosome-carried mHTT propagation is thought to be a novel mechanism for HD pathology, providing a potential therapeutic target for alleviating this neurodegenerative disease (Ananbeh et al., 2021) (Figure 1F).

## Exosomes and Prion Diseases

Prion diseases are a group of neurodegenerative diseases caused by mutated prions. Prions protein-infecting factors, virulent prions or infectious proteins, and the cellular prion protein (PrPc), a cell surface protein encoded by the PRNP gene, is most abundantly expressed in the nervous system, and its misfolded isomer PrP (PrPSc) is key to the development of prion diseases (Ryskalin et al., 2019; Lopez-Perez et al., 2020).

There is plenty of evidence that supports the intercellular transfer of prion proteins *via* exosomes (Cheng et al., 2018). Cellular prion protein (Prpc) regulates cell adhesion and signaling in the brain (Hartmann et al., 2017). Prpc binds to dynein, muskelin, and KIF5C in exosomes, while muskelin coordinates the bidirectional transport of Prpc between the extracellular space and lysosomes. Accumulation of Prpc on the neuronal surface and in secretory exosomes is increased in Muskelin knockout mice. When researchers injected pathogenic prions into Muskelin knockout mice, they found that the onset of prion disease was accelerated (Heisler et al., 2018). On performing miRNA sequencing of the exosomes released from prion-infected neurons, it was revealed that the expression of let-7b, let-7i, miR-21, miR-222, miR-29b, miR-342-3p, and miR-424 were upregulated, whereas miR-146 expression was downregulated (Bellingham et al., 2012; Boese et al., 2016). However, biomarkers for the diagnosis of prion diseases need to be explored further (Figure 1G).

## Exosomes and Cerebrovascular Diseases

Cerebrovascular diseases are divided into cerebral hemorrhagic diseases and cerebral ischemic diseases according to their pathogenesis. Stroke is a cerebrovascular disease that is characterized by a focal neurological deficit due to impaired brain blood circulation. Energy depletion and a hypoxic state after stroke can lead to neuronal damage, which activates resident glial cells and promotes the invasion of peripheral immune cells into the ischemic area of the brain; these immune cells can further necrotize neurons and exacerbate ischemic brain injury, and likewise promote neuronal repair, differentiation, and neural regeneration (Wang et al., 2020a; Xing and Bai, 2020).

The non-coding RNA in exosomes of stroke patients show significant changes. The level of lnc-CRKL-2 and lnc-NTRK3-4 in the serum exosomes of patients with acute minor stroke are increased, while those of RPS6KA2-AS1 and lnc-CALM1-7 are decreased (Xu et al., 2020). Studies have identified important roles for miRNAs in anti-angiogenic mechanisms and cerebrovascular disease (Xin et al., 2017). Some researchers sequenced the blood exosomes from patients with intracranial atherosclerotic disease (associated with high susceptibility to strokes) who did not respond to intensive medical management and found the specific expression of 10 miRNAs, including miR-122-5p, miR-192-5p, and miR-27b-3p. These miRNAs have the potential to be molecular markers for cerebrovascular diseases (Jiang et al., 2019).

Increasing evidence shows that exosomes can play a therapeutic role in stroke (Mirzaei H. et al., 2018; Jafarzadeh-Esfehani et al., 2020; Rahmani et al., 2020). ADEs can mitigate neuronal damage in mice by inhibiting the autophagy of neurons, suggesting that these exosomes can alleviate ischemic stroke (Pei et al., 2019). A similar study revealed that exosomes from MSCs can alleviate the inflammation of astrocytes stimulated by lipopolysaccharides (LPS) in mice, and exosomes can also mitigate LPS-induced abnormal calcium signaling and mitochondrial dysfunction (Xian et al., 2019). Exosomes from BMSCs with high expression of the chemokine receptor CXCR4 promote proliferation and angiogenesis of microvascular endothelial cells in rats with stroke and exert anti-apoptotic effects *via* the Wnt-3a/β-linked protein pathway (Li et al., 2020b).

Increasing number of miRNAs therapeutic targets have been discovered in exosomes. Researchers have attempted to treat stroke by improving the hypoxic state of neuronal cells, promoting vascular regeneration, and modulating the inflammatory response (Chamorro et al., 2021). A recent study has uncovered that exosomes derived from mouse brain vascular endothelial cells can deliver higher levels of miR-126 that can be used to treat mice stroke models with type 2 diabetes, and ease their cognitive function and inflammatory response (Rahmani et al., 2020). Additionally, endothelium-derived exosomes containing miR-126 enrichment are more therapeutically effective than exosomes without miR-126 enrichment (Venkat et al., 2019; Ueno et al., 2020). The exosomes produced by astrocytes carry miR-190b and inhibit the autophagy of nerve cells (from the mouse hippocampus) that are deprived of oxygen and glucose by targeting Atg7 (Pei et al., 2020). In an *in vitro* experiment, the targeting of transient receptor potential melastatin 7 resulted in ADSC-derived exosomal miR-181b-5p inducing an increase in the levels of hypoxia-inducible factor 1α and VEGF, while decreasing the protein expression of the tissue inhibitor of metalloproteinase 3, thereby improving angiogenesis (Yang et al., 2018). BMSCs secrete exosomes loaded with miR-134 that targets caspase-8 to prevent rat oligodendrocyte apoptosis *in vitro*, and it might be a new potential therapeutic target for the treatment of ischemic stroke (Xiao et al., 2018). Exosomes derived from human urine-derived stem cells carry miR-26a (Ling et al., 2020). Researchers injected them into the vein of mice stroke models and found that they can promote functional recovery of stroke by inhibiting histone deacetylase 6 *via* miR-26a (Ling et al., 2020). Secretion of exosomes in multipotent mesenchymal stromal cells transfected with miR-17-92 enhances axonal-myelin remodeling and electrophysiological recovery in mice when injected intravenously, probably due to the downregulation of PTEN by miR-17-92 leading to the activation of the PI3K/Akt/mTOR pathway (Xin et al., 2021) (Figure 1H).

A few studies have attempted to treat an animal stroke model using manually assembled exosomes. They constructed RVG-exosomes loaded with HMGBM1 (high-mobility group box 1) -siRNA and delivered them into the ischemic brain of animal models by intravenous administration. This method could alleviate the inflammation associated with stroke (Kim et al., 2019). The use of macrophage-derived exosomes to deliver edaravone makes it easier to reach ischemic sites. Furthermore, the use of exosomes to deliver edaravone significantly increases its bioavailability, prolongs its half-life, and enhances its original therapeutic effects (Li F. et al., 2020).

## Exosomes and Traumatic Brain Injury

Traumatic brain injury (TBI), also referred to as brain injury or head injury, is a kind of brain tissue damage caused by trauma (Beard et al., 2020).

In recent years, the changes in the exosomal content during the development of disease in patients with TBI have been extensively studied. Brain-injury biomarkers were detected in the CSF exosomes of patients with TBI, such as αII-spectrin breakdown products (BDPs), glial fibrillary acidic protein and its BDPs, ubiquitin C-terminal hydrolase-L1, synaptophysin, and Alix (Manek et al., 2018). This study found that after the occurrence of TBI, changes in the levels of exosomes and their markers in the plasma or CSF does not just diagnose TBI but also stages patients with TBI (Beard et al., 2020; Peltz et al., 2020). In patients with mild TBI (mTBI), the concentration of neuron-derived exosomes in the plasma is reduced by 45% in the acute phase but not in the chronic phase, and the elevation of neuropathological proteins in these exosomes depicts phase-specificity (Peltz et al., 2020). This study suggested that exosomal proteins differed during different periods of TBI (Goetzl et al., 2019). An updated study on exosomes from 195 army veterans showed that compared to controls without TBI, the number of times the veteran was subjected to mTBI correlated with the NfL levels in plasma exosomes. An increase in the number of years since the most recent trauma was correlated with higher plasma exosomal NfL levels, and an increase in the number of years since the first trauma was also correlated with higher plasma exosomal NfL levels. Therefore, NfL level in plasma exosomes can act as a prognostic biomarker for remote symptoms after mTBI (Guedes et al., 2020).

Some exosomal miRNAs play a protective role in TBI (Zhang et al., 2021). Microglial exosomes with upregulated miR-124-3p can improve the neurodegeneration after repetitive mTBI. Microglia have a dual role in the inflammatory response after TBI, inducing a rapid shift from M1 to M2 microglia after the start of the recovery process or promoting microglia M2 polarization, which can suppress the brain inflammatory response and improve neuroprognosis. Microglia exhibited M1 pro-inflammatory phenotype and M2 anti-inflammatory phenotype. miRNA microarray analysis revealed that the expression level of miR-124-3p was most significantly increased. In the TBI mouse model, exosomal miR-124-3p levels gradually increased from the acute to chronic phase. The upregulated exosomal miR-124-3p derived from microglial cells improved the neurodegeneration after repetitive mTBI, promoted microglia anti-inflammatory M2 polarization, inhibited neuronal inflammation, and promoted axonal growth by targeting Rela, which is an inhibitory transcription factor for apolipoprotein E (ApoE) (Huang S. et al., 2018; Li et al., 2019). Exosomal miR-124-3p has been considered a potential treatment option for TBI, and various studies have explored its therapeutic effects (Ge et al., 2020). Recent studies proved that miR-21-5p containing exosomes secreted by neurons mitigate neuroinflammation after TBI by boosting microglial M2 polarization (Yin et al., 2020). miR-873a-5p carried by ADEs inhibits neuroinflammation by inhibiting the NF-κB signaling pathway of neurons after TBI (Long et al., 2020). Researchers co-cultured exosomes from microglia with artificially stretched neurons *in vitro*, while *in vivo* exosomes were administered into the tail vein of mice that had undergone fluid shock damage. The results showed that exosomes from the microglia were absorbed, the dendritic complexity of exosome-treated injured neurons was reduced *in vivo* and *in vitro*, motor function in mice was improved, and the protein levels of GAP43, PSD-95, GluR1, and synaptophysin were reduced in the neurons *in vitro*. However, exosomes produced by the stretch-injured microglia were found to impair motor coordination in TBI mice, which was largely associated with decreased miR-5121 in the exosomes (Zhao et al., 2021).

Meanwhile, the nucleic acids and proteins carried by exosomes can enter the BBB to exert therapeutic effects (Andjus et al., 2020). Damage to the BBB by TBI can be repaired by exosomes derived from human umbilical cord blood-derived endothelial colony-forming cells. These exosomes can also promote the migration of tissue-resident endothelial cells and reduce PTEN expression in endothelial cells incubated under hypoxic conditions, as well as increase AKT phosphorylation and tight junction protein expression (Gao et al., 2018). The exosomes of ADSCs contain MALAT1, a long-chain non-coding RNA, which is required for regulating the cell cycle, cell death, regenerative molecular pathways, and expression of snoRNAs, and is capable of significantly restoring the motor function in mice and reducing cortical brain damage (Patel et al., 2018). ADEs contain GJA1 (gap junction Alpha 1)-20k which they deliver to TBI neurons, thereby decreasing the apoptosis rate, increasing mitochondrial function, and alleviating neuron damage (Chen et al., 2019a). Swine models of TBI that were administered early single-dose exosomes shed from human MSCs showed reduced brain swelling, decreased lesion size, and improved BBB integrity (Williams et al., 2020).

Researchers have utilized modified exosomes to alleviate the symptoms of TBI. Exosomes incorporating plasmids expressing Bcl-2 and Bax shRNA (which can cause Bcl-2 overexpression and inhibit Bax expression) can reduce the levels of Mcl-1, XIAP, and survivin proteins in the brain and release cytochrome C from the mitochondria. Meanwhile, they can also reduce the damage to miniature excitatory postsynaptic current in mice and LTP after TBI, and enhance the motor and cognitive behavior of mice (Wang and Han, 2019) (Figure 1I).

In short, there is sufficient evidence to assert the therapeutic role of exosomes in stroke and TBI. However, before practical clinical applications, the mechanisms by which the exosomes participate in treatment and their exact contents need to be further elucidated. Multiple exosome-related human trials related to the use of exosomes for transporting drugs in stroke models should be performed in the future.

## Exosomes and Mental Disease

Depression, schizophrenia (SCZ), bipolar disorder (BD), autism, etc. comprise the mental diseases discussed in this section.

Intravenous injection of blood exosomes from patients with major depression into the tail of healthy mice causes them to show depression-like behaviors. It has been shown that this effect is mediated by hsa-miR-139-5p (which decreases hippocampal neurogenesis) in the exosomes (Wei et al., 2020). Exosomal miR-207 derived from natural killer cells alleviates the symptoms of depression in mice by targeting the TLR4 interaction with leucine-rich repeats and decreasing NF-κB signaling of astrocytes (Li D. et al., 2020). In one study, brain-derived neurotrophic factor (BDNF) in the serum exosomes of the experimental group (patients with major depression) was significantly reduced compared to healthy controls; however, after 7 weeks of antidepressant treatment, BDNF in the serum exosomes of patients in the experimental group was not significantly different from that in healthy controls. Contrastingly, pro-BDNF was higher in the experimental group compared to the control group before treatment, but was not significantly different after treatment. This study suggests that BDNF may be an effective biomarker for the treatment of depression (Gelle et al., 2021). Compared to healthy controls, the number of L1CAM rich exosomes was increased in patients with major depressive disorder (MDD), and these patients had increased concentrations of insulin receptor substrate −1 (IRS-1) in L1CAM+ exosomes, which is associated with suicidality and anhedonia. Sex differences were observed in serine-312 phosphorylation of IRS-1 in L1CAM+ exosomes of patients with MDD. These findings may provide a basis for the effective treatment of MDD (Nasca et al., 2020).

The miRNA sequencing of plasma exosomes from BD patients and healthy individuals identified 13 abnormal miRNAs. Among them, the level of miR-484, miR-652-3p, and miR-142-3p were significantly decreased, while that of miR-185-5p was significantly increased (Ceylan et al., 2020). On transplanting exosomes secreted by human BMSCs into the lateral ventricles of BTBR mice, their autism-like behavior was reported to be attenuated. These exosomes were found to be capable of ameliorating the symptoms of autism spectrum disorder effectively by nasal injection (Perets et al., 2018). The Shank3B knockout model of autism was treated by intranasal administration of exosomes secreted from MSCs, and after 3 weeks of treatment, it was found that the mice had improved social behavior, increased vocalization, and reduced repetitive behaviors (Perets et al., 2020). This finding may be useful in patients with Shank3B-deficient autism (Perets et al., 2020). Additionally, miR-206, which suppresses the expression of BDNF mRNA and protein, and is used as a latent biomarker for SCZ, was found to be significantly up-regulated in the blood exosomes of patients with SCZ (Du et al., 2019).

Despite numerous studies on the mechanisms of these mental diseases, few have investigated the role of exosomes in depth.

## Exosomes and Epilepsy

Epilepsy is a chronic disease in which sudden abnormal discharges of neurons in the brain lead to transient brain dysfunction (Perucca et al., 2020).

Researchers have found that 42 exosomal miRNAs are differentially expressed in patients with mesial temporal lobe epilepsy with hippocampal sclerosis. Among them, hsa-miR-129-5p,−214-3p,−219a-5p, and−34c-5p are increased, while hsa-miR-421 and−184 are decreased. These aberrantly expressed miRNAs can be used as potential targets for disease diagnosis and treatment (Chen S. D. et al., 2020; Huang et al., 2020). Measurement of proteins in serum exosomes from patients with epilepsy revealed that coagulation factor IX (F9) and thrombospondin-1 represent potential new markers for the diagnosis of epilepsy (Lin et al., 2020). This was the first time that exosomal proteins have been measured in epileptic patients, and conducting further exosomal studies in the field of epilepsy is essential (Lin et al., 2020) (Figure 1J).

## Exosomes and Meningitis

Meningitis, which is caused by multiple biological pathogenic factors invading the pia mater and spinal membranes, is considered a diffuse inflammation of the meninges. Long-term sequelae comprise the primary concern during the treatment of this disease.

Researchers have shown that proteins that take part in the immune response and exosome signal transduction are enriched in the CSF of patients with streptococcal meningitis, supporting the potential role of exosomes in the progression of meningitis. Exosomes can potentially provide a non-invasive and accurate method for detecting variations in the central nervous system after meningitis, and guide optimal treatment. However, little relevant research has been undertaken thus far in this area (Gomez-Baena et al., 2017).

## Future Perspectives

Exosomes are inextricably linked to the progression of nervous system disease, as they can convey pathological proteins to various neurons and accelerate the progression of disease. Exosomes are also involved in the self-rescue of neurons, and neurons can remove detrimental substances by the secretion of exosomes. Nevertheless, whether exosomes allow neurons to save themselves or transmit proteins to other neurons to resulting in more serious consequences, needs to be explored further.

Exosomes have been used as a diagnostic and treatment tool in animal experiments. Due to their ability to reflect the course of the disease, exosomes in the blood, CSF, urine, and saliva, which contain diverse biomarkers, are convenient and non-invasive tools for the early detection of diseases as well as for developing therapeutic strategies. Recent studies have shown that Aβ42, T-tau, and P-T181-tau in blood exosomes can be used to diagnose AD and amnestic mild cognitive impairment (Jia et al., 2019). Exosomes can also serve as carriers for drug delivery, and some studies have modified their surface to improve their targeting ability, which enables better drug absorption compared to the traditional routes of administration. Compared to a direct injection of MSCs, exosomes can pass through the BBB and minimize immune rejection, leading to improved drug absorption and treatment in patients with AD or PD (Jin et al., 2021).

However, there are some problems that need to be conclusively resolved in this field. Although exosomes transporting drugs were found to be fully absorbed by the target cells *in vitro* and achieved the desired results, whether this effect is the same *in vivo*, and is not associated with any side-effects, is an issue that still needs further exploration. The artificial synthesis of exosome-like nanovesicles with the retention of the key exosome molecules can help avoid the disadvantages mentioned above and make better use of exosomes; moreover, this has become a research hotspot (Lu and Huang, 2020). Furthermore, the modification of the exosome surface to increase its targeting ability and the construction of a better exosome separation and purification system have also been attracting research interest recently. Furthermore, prior to using the cargo carried by exosomes as biomarkers for clinical diagnosis, we require more supporting data with higher accuracy. Beyond this, the relationship between the abnormal rise or decline of biomarkers and disease progression needs further study, and it is hoped that exosomes can provide a foundation for clinical staging of certain diseases.

## Author Contributions

LZ proposed and revised the manuscript. NZ and FH co-wrote this manuscript. All authors reviewed the manuscript and approved of the final version.

## Funding

This work was supported by grants from the National Natural Science Foundation of China (No. 81903030), Outstanding Young Aid Program for Education Department of Hunan Province (Grant No. 18B274), the Natural Science Foundation of Hunan Province, China (Nos. S2021JJQNJJ1153, 2019JJ40249, and 2018JJ3455), Cooperative Education Program of Ministry of Education (No. 202002138007), and Key Project of Hunan Provincial Department of Education (20A427).

## Conflict of Interest

The authors declare that the research was conducted in the absence of any commercial or financial relationships that could be construed as a potential conflict of interest.

## Publisher's Note

All claims expressed in this article are solely those of the authors and do not necessarily represent those of their affiliated organizations, or those of the publisher, the editors and the reviewers. Any product that may be evaluated in this article, or claim that may be made by its manufacturer, is not guaranteed or endorsed by the publisher.

## Abbreviations

EVs: Extracellular vesicles
Alix: ALG2-interacting protein X
APP: amyloid precursor protein
α-syn: α-synuclein
BBB: blood-brain barrier
GBM: glioblastoma
mTOR: mammalian target of rapamycin
CSF: cerebrospinal fluid
TAM: tumor-associated macrophages
L1CAM: cell adhesion molecule L1
PTRF: transcript release factor
miRNA: microRNA
KLF2: Kruppel-like factors 2
KLF4: Kruppel-like factors 4
VEGF: vascular endothelial growth factor
LncRNA: long non-coding RNA
TMZ: Temozolomide
BMSCs: bone marrow-derived mesenchymal stem cells
AD: Alzheimer's disease
Aβ: Amyloid beta
iPSC: induced pluripotent stem cell
mTau: mutant tau
ANP32A: acidic nuclear phosphoprotein 32 family member A
ADEs: astrocyte-derived exosomes
CR1: complement receptor type 1
GAC: glutaminase C
piRNAs: PIWI-interacting RNAs
GAP43: growth associated protein 43
MSCs: mesenchymal stem cells
LTP: long-term potentiation
CA1: cornu ammonis 1
PD: Parkinson's disease
DA: dopaminergic
ASO: antisense oligonucleotides
ALS: Amyotrophic lateral sclerosis
SOD1: superoxide dismutase 1
TDP: TDR DNA-binding protein
IL: Interleukin
NIR: INHAT repressor
ADSCs: adipose-derived stem cells
MS: Multiple sclerosis
IGF1R: growth factor 1 receptor
PBMC: peripheral mononuclear blood cells
HD: Huntington's disease
Prpc: cellular prion protein
LPS: lipopolysaccharides
HMGBM1: high-mobility group box 1
TBI: Traumatic brain injury
BDPs: breakdown products
mTBI: mild TBI
ApoE: apolipoprotein E
GJA1: gap junction Alpha 1
SCZ: schizophrenia
BD: bipolar disorder
BDNF: brain-derived neurotrophic factor
MDD: major depressive disorder
IRS-1: insulin receptor substrate−1.

## References

[B1] Agliardi C. Guerini F. R. Zanzottera M. Bianchi A. Nemni R. Clerici M. (2019). SNAP-25 in serum is carried by exosomes of neuronal origin and is a potential biomarker of Alzheimer's disease. Mol. Neurobiol. 56, 5792–5798. 10.1007/s12035-019-1501-x 30680692

[B2] Ananbeh H. Vodicka P. Kupcova Skalnikova H. (2021). Emerging roles of exosomes in Huntington's disease. Int. J. Mol. Sci. 22:4085. 10.3390/ijms22084085 33920936 PMC8071291

[B3] Andjus P. Kosanović M. Milićević K. Gautam M. Vainio S. J. Jagečić D.. (2020). Extracellular vesicles as innovative tool for diagnosis, regeneration and protection against neurological damage. Int. J. Mol. Sci. 21:6859. 10.3390/ijms21186859 32962107 PMC7555813

[B4] Azambuja J. H. Ludwig N. Yerneni S. S. Braganhol E. Whiteside T. L. (2020). Arginase-1+ exosomes from reprogrammed macrophages promote glioblastoma progression. Int. J. Mol. Sci. 21:3990. 10.3390/ijms21113990 32498400 PMC7312363

[B5] Azarmi M. Maleki H. Nikkam N. Malekinejad H. (2020). Transcellular brain drug delivery: a review on recent advancements. Int. J. Pharm. 586:119582. 10.1016/j.ijpharm.2020.119582 32599130

[B6] Baharlooi H. Nouraei Z. Azimi M. Moghadasi A. N. Tavassolifar M. J. Moradi B.. (2021). Umbilical cord mesenchymal stem cells as well as their released exosomes suppress proliferation of activated PBMCs in multiple sclerosis. Scand. J. Immunol. 93:e13013. 10.1111/sji.13013 33338274

[B7] Baig M. S. Roy A. Rajpoot S. Liu D. Savai R. Banerjee S.. (2020). Tumor-derived exosomes in the regulation of macrophage polarization. Inflammation Res. 69, 435–451. 10.1007/s00011-020-01318-0 32162012

[B8] Beard K. Meaney D. F. Issadore D. (2020). Clinical applications of extracellular vesicles in the diagnosis and treatment of traumatic brain injury. J. Neurotrauma 37, 2045–2056. 10.1089/neu.2020.6990 32312151 PMC7502684

[B9] Beatriz M. Vilaca R. Lopes C. (2021). Exosomes: innocent bystanders or critical culprits in neurodegenerative diseases. Front. Cell Dev. Biol. 9:635104. 10.3389/fcell.2021.635104 34055771 PMC8155522

[B10] Beeraka N. M. Doreswamy S. H. Sadhu S. P. Srinivasan A. Pragada R. R. Madhunapantula S. V.. (2020). The role of exosomes in stemness and neurodegenerative diseases-chemoresistant-cancer therapeutics and phytochemicals. Int. J. Mol. Sci. 21:6818. 10.3390/ijms21186818 32957534 PMC7555629

[B11] Bellingham S. A. Coleman B. M. Hill A. F. (2012). Small RNA deep sequencing reveals a distinct miRNA signature released in exosomes from prion-infected neuronal cells. Nucleic Acids Res. 40, 10937–10949. 10.1093/nar/gks832 22965126 PMC3505968

[B12] Boese A. S. Saba R. Campbell K. Majer A. Medina S. Burton L.. (2016). MicroRNA abundance is altered in synaptoneurosomes during prion disease. Mol. Cell. Neurosci. 71, 13–24. 10.1016/j.mcn.2015.12.001 26658803

[B13] Bonafede R. Turano E. Scambi I. Busato A. Bontempi P. Virla F.. (2020). ASC-exosomes ameliorate the disease progression in SOD1(G93A) murine model underlining their potential therapeutic use in human ALS. Int. J. Mol. Sci. 21:3651. 10.3390/ijms21103651 32455791 PMC7279464

[B14] Budnik V. Ruiz-Canada C. Wendler F. (2016). Extracellular vesicles round off communication in the nervous system. Nat. Rev. Neurosci. 17, 160–172. 10.1038/nrn.2015.29 26891626 PMC4989863

[B15] Calabria E. Scambi I. Bonafede R. Schiaffino L. Peroni D. Potrich V.. (2019). ASCs-exosomes recover coupling efficiency and mitochondrial membrane potential in an *in vitro* model of ALS. Front. Neurosci. 13:1070. 10.3389/fnins.2019.01070 31680811 PMC6811497

[B16] Ceylan D. Tufekci K. U. Keskinoglu P. Genc S. Ozerdem A. (2020). Circulating exosomal microRNAs in bipolar disorder. J. Affect. Disord. 262, 99–107. 10.1016/j.jad.2019.10.038 31726266

[B17] Cha D. J. Mengel D. Mustapic M. Liu W. Selkoe D. J. Kapogiannis D.. (2019). miR-212 and miR-132 are downregulated in neurally derived plasma exosomes of Alzheimer's patients. Front. Neurosci. 13:1208. 10.3389/fnins.2019.01208 31849573 PMC6902042

[B18] Chamorro A. Lo E. H. Renu A. Van Leyen K. Lyden P. D. (2021). The future of neuroprotection in stroke. J. Neurol. Neurosurg. Psychiatry 92, 129–135. 10.1136/jnnp-2020-324283 33148815

[B19] Chen P. C. Wu D. Hu C. J. Chen H. Y. Hsieh Y. C. Huang C. C. (2020). Exosomal TAR DNA-binding protein-43 and neurofilaments in plasma of amyotrophic lateral sclerosis patients: a longitudinal follow-up study. J. Neurol. Sci. 418:117070. 10.1016/j.jns.2020.117070 32836016

[B20] Chen S. D. Pan H. Y. Huang J. B. Liu X. P. Li J. H. Ho C. J.. (2020). Circulating MicroRNAs from serum exosomes may serve as a putative biomarker in the diagnosis and treatment of patients with focal cortical dysplasia. Cells 9:1867. 10.3390/cells9081867 32785072 PMC7465068

[B21] Chen W. Zheng P. Hong T. Wang Y. Liu N. He B.. (2019a). Astrocytes-derived exosomes induce neuronal recovery after traumatic brain injury via delivering gap junction alpha 1-20 k. J. Tissue Eng. Regen. Med. 14, 412–423. 10.1002/term.3002 31826322

[B22] Chen Y. Xia K. Chen L. Fan D. (2019b). Increased interleukin-6 levels in the astrocyte-derived exosomes of sporadic amyotrophic lateral sclerosis patients. Front. Neurosci. 13:574. 10.3389/fnins.2019.00574 31231184 PMC6560167

[B23] Cheng L. Zhao W. Hill A. F. (2018). Exosomes and their role in the intercellular trafficking of normal and disease associated prion proteins. Mol. Aspects Med. 60, 62–68. 10.1016/j.mam.2017.11.011 29196098

[B24] Choi Y. Kim S. M. Heo Y. Lee G. Kang J. Y. Yoon D. S. (2021). Nanoelectrical characterization of individual exosomes secreted by Abeta42-ingested cells using electrostatic force microscopy. Nanotechnology 32:025705. 10.1088/1361-6528/abba58 32957091

[B25] Cui G. H. Wu J. Mou F. F. Xie W. H. Wang F. B. Wang Q. L.. (2018). Exosomes derived from hypoxia-preconditioned mesenchymal stromal cells ameliorate cognitive decline by rescuing synaptic dysfunction and regulating inflammatory responses in APP/PS1 mice. FASEB J. 32, 654–668. 10.1096/fj.201700600R 28970251

[B26] Dai X. Liao K. Zhuang Z. Chen B. Zhou Z. Zhou S.. (2019). AHIF promotes glioblastoma progression and radioresistance via exosomes. Int. J. Oncol. 54, 261–270. 10.3892/ijo.2018.4621 30387845

[B27] De Boer E. M. J. Orie V. K. Williams T. Baker M. R. De Oliveira H. M. Polvikoski T.. (2020). TDP-43 proteinopathies: a new wave of neurodegenerative diseases. J. Neurol. Neurosurg. Psychiatry. 92, 86–95. 10.1136/jnnp-2020-322983 33177049 PMC7803890

[B28] De Leo A. Ugolini A. Veglia F. (2020). Myeloid cells in glioblastoma microenvironment. Cells 10:18. 10.3390/cells10010018 33374253 PMC7824606

[B29] Deng S. Zhou X. Ge Z. Song Y. Wang H. Liu X.. (2019). Exosomes from adipose-derived mesenchymal stem cells ameliorate cardiac damage after myocardial infarction by activating S1P/SK1/S1PR1 signaling and promoting macrophage M2 polarization. Int. J. Biochem. Cell Biol. 114:105564. 10.1016/j.biocel.2019.105564 31276786

[B30] Didiot M. C. Hall L. M. Coles A. H. Haraszti R. A. Godinho B. M. Chase K.. (2016). Exosome-mediated delivery of hydrophobically modified siRNA for Huntingtin mRNA silencing. Mol. Ther. 24, 1836–1847. 10.1038/mt.2016.126 27506293 PMC5112038

[B31] Du Y. Yu Y. Hu Y. Li X. W. Wei Z. X. Pan R. Y.. (2019). Genome-wide, integrative analysis implicates exosome-derived microRNA dysregulation in schizophrenia. Schizophr. Bull. 45, 1257–1266. 10.1093/schbul/sby191 30770930 PMC6811837

[B32] Fernandes A. Ribeiro A. R. Monteiro M. Garcia G. Vaz A. R. Brites D. (2018). Secretome from SH-SY5Y APPSwe cells trigger time-dependent CHME3 microglia activation phenotypes, ultimately leading to miR-21 exosome shuttling. Biochimie 155, 67–82. 10.1016/j.biochi.2018.05.015 29857185

[B33] Galazka G. Mycko M. P. Selmaj I. Raine C. S. Selmaj K. W. (2018). Multiple sclerosis: serum-derived exosomes express myelin proteins. Mult. Scler. 24, 449–458. 10.1177/1352458517696597 28273783

[B34] Gao G. Zhao S. Xia X. Li C. Li C. Ji C.. (2019). Glutaminase C regulates microglial activation and pro-inflammatory exosome release: relevance to the pathogenesis of Alzheimer's disease. Front. Cell. Neurosci. 13:264. 10.3389/fncel.2019.00264 31316350 PMC6611423

[B35] Gao W. Li F. Liu L. Xu X. Zhang B. Wu Y.. (2018). Endothelial colony-forming cell-derived exosomes restore blood-brain barrier continuity in mice subjected to traumatic brain injury. Exp. Neurol. 307, 99–108. 10.1016/j.expneurol.2018.06.001 29883579

[B36] Ge X. Guo M. Hu T. Li W. Huang S. Yin Z.. (2020). Increased microglial exosomal miR-124-3p alleviates neurodegeneration and improves cognitive outcome after rmTBI. Mol. Ther. 28, 503–522. 10.1016/j.ymthe.2019.11.017 31843449 PMC7001001

[B37] Gelle T. Samey R. A. Plansont B. Bessette B. Jauberteau-Marchan M. O. Lalloue F.. (2021). BDNF and pro-BDNF in serum and exosomes in major depression: evolution after antidepressant treatment. Prog. Neuropsychopharmacol. Biol. Psychiatry 109:110229. 10.1016/j.pnpbp.2020.110229 33358963

[B38] Giunti D. Marini C. Parodi B. Usai C. Milanese M. Bonanno G.. (2021). Role of miRNAs shuttled by mesenchymal stem cell-derived small extracellular vesicles in modulating neuroinflammation. Sci. Rep. 11:1740. 10.1038/s41598-021-81039-4 33462263 PMC7814007

[B39] Goetzl E. J. Elahi F. M. Mustapic M. Kapogiannis D. Pryhoda M. Gilmore A.. (2019). Altered levels of plasma neuron-derived exosomes and their cargo proteins characterize acute and chronic mild traumatic brain injury. FASEB J. 33, 5082–5088. 10.1096/fj.201802319R 30605353 PMC6436652

[B40] Goetzl E. J. Schwartz J. B. Abner E. L. Jicha G. A. Kapogiannis D. (2018). High complement levels in astrocyte-derived exosomes of Alzheimer disease. Ann. Neurol. 83, 544–552. 10.1002/ana.25172 29406582 PMC5867263

[B41] Gomez-Baena G. Bennett R. J. Martinez-Rodriguez C. Wnek M. Laing G. Hickey G.. (2017). Quantitative proteomics of cerebrospinal fluid in paediatric pneumococcal meningitis. Sci. Rep. 7:7042. 10.1038/s41598-017-07127-6 28765563 PMC5539295

[B42] Guedes V. A. Kenney K. Shahim P. Qu B. X. Lai C. Devoto C.. (2020). Exosomal neurofilament light: a prognostic biomarker for remote symptoms after mild traumatic brain injury? Neurology 94, e2412–e2423. 10.1212/WNL.0000000000009577 32461282 PMC7455370

[B43] Guo M. Wang J. Zhao Y. Feng Y. Han S. Dong Q.. (2020). Microglial exosomes facilitate alpha-synuclein transmission in Parkinson's disease. Brain 143, 1476–1497. 10.1093/brain/awaa090 32355963 PMC7241957

[B44] Hartmann A. Muth C. Dabrowski O. Krasemann S. Glatzel M. (2017). Exosomes and the prion protein: more than one truth. Front. Neurosci. 11:194. 10.3389/fnins.2017.00194 28469550 PMC5395619

[B45] Hayashi N. Doi H. Kurata Y. Kagawa H. Atobe Y. Funakoshi K.. (2019). Proteomic analysis of exosome-enriched fractions derived from cerebrospinal fluid of amyotrophic lateral sclerosis patients. Neurosci. Res. 160, 43–49. 10.1016/j.neures.2019.10.010 31669371

[B46] He M. Zhang H. N. Tang Z. C. Gao S. G. (2021a). Diagnostic and therapeutic potential of exosomal microRNAs for neurodegenerative diseases. Neural Plast. 2021:8884642. 10.1155/2021/8884642 34054944 PMC8143892

[B47] He X. Kuang G. Wu Y. Ou C. (2021b). Emerging roles of exosomal miRNAs in diabetes mellitus. Clin. Transl. Med. 11:e468. 10.1002/ctm2.468 34185424 PMC8236118

[B48] Heisler F. F. Pechmann Y. Wieser I. Altmeppen H. C. Veenendaal L. Muhia M.. (2018). Muskelin coordinates PrP(C) lysosome versus exosome targeting and impacts prion disease progression. Neuron 99, 1155–1169 e1159. 10.1016/j.neuron.2018.08.010 30174115

[B49] Hill A. F. (2019). Extracellular vesicles and neurodegenerative diseases. J. Neurosci. 39, 9269–9273. 10.1523/jneurosci.0147-18.2019 31748282 PMC6867808

[B50] Huang K. Fang C. Yi K. Liu X. Qi H. Tan Y.. (2018). The role of PTRF/Cavin1 as a biomarker in both glioma and serum exosomes. Theranostics 8, 1540–1557. 10.7150/thno.22952 29556340 PMC5858166

[B51] Huang L. G. Luo Y. H. Xu J. W. Lu Q. C. (2020). Plasma exosomal MiRNAs expression profile in mesial temporal lobe epilepsy with hippocampal sclerosis: case-control study and analysis of potential functions. Front. Mol. Neurosci. 13:584828. 10.3389/fnmol.2020.584828 33240042 PMC7680973

[B52] Huang S. Ge X. Yu J. Han Z. Yin Z. Li Y.. (2018). Increased miR-124-3p in microglial exosomes following traumatic brain injury inhibits neuronal inflammation and contributes to neurite outgrowth via their transfer into neurons. FASEB J. 32, 512–528. 10.1096/fj.201700673R 28935818

[B53] Iguchi Y. Eid L. Parent M. Soucy G. Bareil C. Riku Y.. (2016). Exosome secretion is a key pathway for clearance of pathological TDP-43. Brain 139(Pt 12), 3187–3201. 10.1093/brain/aww237 27679482 PMC5840881

[B54] Izco M. Blesa J. Schleef M. Schmeer M. Porcari R. Al-Shawi R.. (2019). Systemic exosomal delivery of shRNA minicircles prevents parkinsonian pathology. Mol. Ther. 27, 2111–2122. 10.1016/j.ymthe.2019.08.010 31501034 PMC6904801

[B55] Jackle K. Zeis T. Schaeren-Wiemers N. Junker A. Van Der Meer F. Kramann N.. (2020). Molecular signature of slowly expanding lesions in progressive multiple sclerosis. Brain 143, 2073–2088. 10.1093/brain/awaa158 32577755

[B56] Jafari D. Shajari S. Jafari R. Mardi N. Gomari H. Ganji F.. (2020). Designer exosomes: a new platform for biotechnology therapeutics. BioDrugs 34, 567–586. 10.1007/s40259-020-00434-x 32754790 PMC7402079

[B57] Jafarzadeh-Esfehani R. Soudyab M. Parizadeh S. M. Jaripoor M. E. Nejad P. S. Shariati M.. (2020). Circulating exosomes and their role in stroke. Curr. Drug Targets 21, 89–95. 10.2174/1389450120666190821153557 31433753

[B58] Jahangard Y. Monfared H. Moradi A. Zare M. Mirnajafi-Zadeh J. Mowla S. J. (2020). Therapeutic effects of transplanted exosomes containing miR-29b to a rat model of Alzheimer's disease. Front. Neurosci. 14:564. 10.3389/fnins.2020.00564 32625049 PMC7314926

[B59] Jain G. Stuendl A. Rao P. Berulava T. Pena Centeno T. Kaurani L.. (2019). A combined miRNA-piRNA signature to detect Alzheimer's disease. Transl. Psychiatry 9:250. 10.1038/s41398-019-0579-2 31591382 PMC6779890

[B60] Jeon I. Cicchetti F. Cisbani G. Lee S. Li E. Bae J.. (2016). Human-to-mouse prion-like propagation of mutant huntingtin protein. Acta Neuropathol. 132, 577–592. 10.1007/s00401-016-1582-9 27221146 PMC5023734

[B61] Jia L. Qiu Q. Zhang H. Chu L. Du Y. Zhang J.. (2019). Concordance between the assessment of Abeta42, T-tau, and P-T181-tau in peripheral blood neuronal-derived exosomes and cerebrospinal fluid. Alzheimers. Dement. 15, 1071–1080. 10.1016/j.jalz.2019.05.002 31422798

[B62] Jia L. Zhu M. Kong C. Pang Y. Zhang H. Qiu Q.. (2021). Blood neuro-exosomal synaptic proteins predict Alzheimer's disease at the asymptomatic stage. Alzheimers. Dement. 17, 49–60. 10.1002/alz.12166 32776690 PMC7984076

[B63] Jiang C. Hopfner F. Katsikoudi A. Hein R. Catli C. Evetts S.. (2020). Serum neuronal exosomes predict and differentiate Parkinson's disease from atypical parkinsonism. J Neurol. Neurosurg. Psychiatry 91, 720–729. 10.1136/jnnp-2019-322588 32273329 PMC7361010

[B64] Jiang H. Toscano J. F. Song S. S. Schlick K. H. Dumitrascu O. M. Pan J.. (2019). Differential expression of circulating exosomal microRNAs in refractory intracranial atherosclerosis associated with antiangiogenesis. Sci. Rep. 9:19429. 10.1038/s41598-019-54542-y 31857618 PMC6923371

[B65] Jin Q. Wu P. Zhou X. Qian H. Xu W. (2021). Extracellular vesicles: novel roles in neurological disorders. Stem Cells Int. 2021:6640836. 10.1155/2021/6640836 33679989 PMC7904361

[B66] Kim E. Otgontenger U. Jamsranjav A. Kim S. S. (2020). Deleterious alteration of glia in the brain of Alzheimer's disease. Int. J. Mol. Sci. 21:6676. 10.3390/ijms21186676 32932623 PMC7555758

[B67] Kim M. Kim G. Hwang D. W. Lee M. (2019). Delivery of high mobility group box-1 siRNA using brain-targeting exosomes for ischemic stroke therapy. J. Biomed. Nanotechnol. 15, 2401–2412. 10.1166/jbn.2019.2866 31748020

[B68] Kimura K. Hohjoh H. Fukuoka M. Sato W. Oki S. Tomi C.. (2018). Circulating exosomes suppress the induction of regulatory T cells via let-7i in multiple sclerosis. Nat. Commun. 9:17. 10.1038/s41467-017-02406-2 29295981 PMC5750223

[B69] Leblanc P. Arellano-Anaya Z. E. Bernard E. Gallay L. Provansal M. Lehmann S.. (2017). Isolation of exosomes and microvesicles from cell culture systems to study prion transmission. Methods Mol. Biol. 1545, 153–176. 10.1007/978-1-4939-6728-5_11 27943213

[B70] Lee M. Im W. Kim M. (2021). Exosomes as a potential messenger unit during heterochronic parabiosis for amelioration of Huntington's disease. Neurobiol. Dis. 155:105374. 10.1016/j.nbd.2021.105374 33940179

[B71] Lee M. Liu T. Im W. Kim M. (2016). Exosomes from adipose-derived stem cells ameliorate phenotype of Huntington's disease *in vitro* model. Eur. J. Neurosci. 44, 2114–2119. 10.1111/ejn.13275 27177616

[B72] Li B. Liu J. Gu G. Han X. Zhang Q. Zhang W. (2020). Impact of neural stem cell-derived extracellular vesicles on mitochondrial dysfunction, sirtuin 1 level, and synaptic deficits in Alzheimer's disease. J. Neurochem. 154, 502–518. 10.1111/jnc.15001 32145065

[B73] Li D. Huang S. Yin Z. Zhu J. Ge X. Han Z.. (2019). Increases in miR-124-3p in microglial exosomes confer neuroprotective effects by targeting FIP200-mediated neuronal autophagy following traumatic brain injury. Neurochem. Res. 44, 1903–1923. 10.1007/s11064-019-02825-1 31190315

[B74] Li D. Wang Y. Jin X. Hu D. Xia C. Xu H.. (2020). NK cell-derived exosomes carry miR-207 and alleviate depression-like symptoms in mice. J. Neuroinflammation 17:126. 10.1186/s12974-020-01787-4 32321532 PMC7178582

[B75] Li F. Zhao L. Shi Y. Liang J. (2020). Edaravone-loaded macrophage-derived exosomes enhance neuroprotection in the rat permanent middle cerebral artery occlusion model of stroke. Mol. Pharm. 17, 3192–3201. 10.1021/acs.molpharmaceut.0c00245 32786956

[B76] Li J. Yuan H. Xu H. Zhao H. Xiong N. (2020). Hypoxic cancer-secreted exosomal miR-182-5p promotes glioblastoma angiogenesis by targeting kruppel-like factor 2 and 4. Mol. Cancer Res. 18, 1218–1231. 10.1158/1541-7786.MCR-19-0725 32366676

[B77] Li X. Zhang J. Zhang X. Dong M. (2020a). Puerarin suppresses MPP(+)/MPTP-induced oxidative stress through an Nrf2-dependent mechanism. Food Chem Toxicol. 144:111644. 10.1016/j.fct.2020.111644 32763437

[B78] Li X. Zhang Y. Wang Y. Zhao D. Sun C. Zhou S.. (2020b). Exosomes derived from CXCR4-overexpressing BMSC promoted activation of microvascular endothelial cells in cerebral ischemia/reperfusion injury. Neural Plast. 2020:8814239. 10.1155/2020/8814239 33381162 PMC7762674

[B79] Lin Z. Gu Y. Zhou R. Wang M. Guo Y. Chen Y.. (2020). Serum exosomal proteins F9 and TSP-1 as potential diagnostic biomarkers for newly diagnosed epilepsy. Front. Neurosci. 14:737. 10.3389/fnins.2020.00737 32848539 PMC7417627

[B80] Ling X. Zhang G. Xia Y. Zhu Q. Zhang J. Li Q.. (2020). Exosomes from human urine-derived stem cells enhanced neurogenesis via miR-26a/HDAC6 axis after ischaemic stroke. J. Cell. Mol. Med. 24, 640–654. 10.1111/jcmm.14774 31667951 PMC6933407

[B81] Long X. Yao X. Jiang Q. Yang Y. He X. Tian W.. (2020). Astrocyte-derived exosomes enriched with miR-873a-5p inhibit neuroinflammation via microglia phenotype modulation after traumatic brain injury. J. Neuroinflammation 17:89. 10.1186/s12974-020-01761-0 32192523 PMC7082961

[B82] Lopez-Perez O. Badiola J. J. Bolea R. Ferrer I. Llorens F. Martin-Burriel I. (2020). An update on autophagy in prion diseases. Front Bioeng Biotechnol. 8:975. 10.3389/fbioe.2020.00975 32984276 PMC7481332

[B83] Losurdo M. Pedrazzoli M. D'agostino C. Elia C. A. Massenzio F. Lonati E.. (2020). Intranasal delivery of mesenchymal stem cell-derived extracellular vesicles exerts immunomodulatory and neuroprotective effects in a 3xTg model of Alzheimer's disease. Stem Cells Transl. Med. 9, 1068–1084. 10.1002/sctm.19-0327 32496649 PMC7445021

[B84] Lu M. Huang Y. (2020). Bioinspired exosome-like therapeutics and delivery nanoplatforms. Biomaterials 242:119925. 10.1016/j.biomaterials.2020.119925 32151860

[B85] Luciunaite A. Mcmanus R. M. Jankunec M. Racz I. Dansokho C. Dalgediene I.. (2020). Soluble Abeta oligomers and protofibrils induce NLRP3 inflammasome activation in microglia. J. Neurochem. 155, 650–661. 10.1111/jnc.14945 31872431

[B86] Manek R. Moghieb A. Yang Z. Kumar D. Kobeissy F. Sarkis G. A.. (2018). Correction to: protein biomarkers and neuroproteomics characterization of microvesicles/exosomes from human cerebrospinal fluid following traumatic brain injury. Mol. Neurobiol. 55:6129. 10.1007/s12035-018-0909-z 29344927

[B87] Martinez B. Peplow P. V. (2020). MicroRNAs as disease progression biomarkers and therapeutic targets in experimental autoimmune encephalomyelitis model of multiple sclerosis. Neural Regen Res. 15, 1831–1837. 10.4103/1673-5374.280307 32246624 PMC7513985

[B88] Mckeever P. M. Schneider R. Taghdiri F. Weichert A. Multani N. Brown R. A.. (2018). MicroRNA expression levels are altered in the cerebrospinal fluid of patients with young-onset Alzheimer's disease. Mol. Neurobiol. 55, 8826–8841. 10.1007/s12035-018-1032-x 29603092 PMC6208843

[B89] Micci M. A. Krishnan B. Bishop E. Zhang W. R. Guptarak J. Grant A.. (2019). Hippocampal stem cells promotes synaptic resistance to the dysfunctional impact of amyloid beta oligomers via secreted exosomes. Mol. Neurodegener. 14:25. 10.1186/s13024-019-0322-8 31200742 PMC6570890

[B90] Mirzaei H. Momeni F. Saadatpour L. Sahebkar A. Goodarzi M. Masoudifar A.. (2018). MicroRNA: relevance to stroke diagnosis, prognosis, and therapy. J. Cell. Physiol. 233, 856–865. 10.1002/jcp.25787 28067403

[B91] Mirzaei R. Sarkar S. Dzikowski L. Rawji K. S. Khan L. Faissner A.. (2018). Brain tumor-initiating cells export tenascin-C associated with exosomes to suppress T cell activity. Oncoimmunology 7:e1478647. 10.1080/2162402X.2018.1478647 30288344 PMC6169571

[B92] Munoz J. L. Walker N. D. Mareedu S. Pamarthi S. H. Sinha G. Greco S. J.. (2019). Cycling quiescence in temozolomide resistant glioblastoma cells is partly explained by microRNA-93 and−193-mediated decrease of cyclin D. Front. Pharmacol. 10:134. 10.3389/fphar.2019.00134 30853911 PMC6395452

[B93] Nasca C. Dobbin J. Bigio B. Watson K. De Angelis P. Kautz M.. (2020). Insulin receptor substrate in brain-enriched exosomes in subjects with major depression: on the path of creation of biosignatures of central insulin resistance. Mol. Psychiatry. 10.1038/s41380-020-0804-7. [Epub ahead of print]. 32536688 PMC7787430

[B94] Nieland L. Morsett L. M. Broekman M. L. D. Breakefield X. O. Abels E. R. (2021). Extracellular vesicle-mediated bilateral communication between glioblastoma and astrocytes. Trends Neurosci. 44, 215–226. 10.1016/j.tins.2020.10.014 33234347 PMC7904598

[B95] Otake K. Kamiguchi H. Hirozane Y. (2019). Identification of biomarkers for amyotrophic lateral sclerosis by comprehensive analysis of exosomal mRNAs in human cerebrospinal fluid. BMC Med. Genomics. 12:7. 10.1186/s12920-019-0473-z 30630471 PMC6329125

[B96] Ou A. Yung W. K. A. Majd N. (2020). Molecular mechanisms of treatment resistance in glioblastoma. Int. J. Mol. Sci. 22. 10.3390/ijms22010351 33396284 PMC7794986

[B97] Pace K. R. Dutt R. Galileo D. S. (2019). Exosomal L1CAM stimulates glioblastoma cell motility, proliferation, and invasiveness. Int. J. Mol. Sci. 20:3982. 10.3390/ijms20163982 31426278 PMC6720723

[B98] Pan J. He R. Huo Q. Shi Y. Zhao L. (2020). Brain microvascular endothelial cell derived exosomes potently ameliorate cognitive dysfunction by enhancing the clearance of abeta through up-regulation of P-gp in mouse model of AD. Neurochem. Res. 45, 2161–2172. 10.1007/s11064-020-03076-1 32583212

[B99] Park G. Kim B. S. Kim E. (2020). A novel function of FAF1, which induces dopaminergic neuronal death through cell-to-cell transmission. Cell Commun. Signal. 18:133. 10.1186/s12964-020-00632-8 32831099 PMC7444258

[B100] Patel N. A. Moss L. D. Lee J. Y. Tajiri N. Acosta S. Hudson C.. (2018). Long noncoding RNA MALAT1 in exosomes drives regenerative function and modulates inflammation-linked networks following traumatic brain injury. J. Neuroinflammation 15:204. 10.1186/s12974-018-1240-3 30001722 PMC6044101

[B101] Pei X. Li Y. Zhu L. Zhou Z. (2019). Astrocyte-derived exosomes suppress autophagy and ameliorate neuronal damage in experimental ischemic stroke. Exp. Cell Res. 382:111474. 10.1016/j.yexcr.2019.06.019 31229506

[B102] Pei X. Li Y. Zhu L. Zhou Z. (2020). Astrocyte-derived exosomes transfer miR-190b to inhibit oxygen and glucose deprivation-induced autophagy and neuronal apoptosis. Cell Cycle 19, 906–917. 10.1080/15384101.2020.1731649 32150490 PMC7217362

[B103] Peltz C. B. Kenney K. Gill J. Diaz-Arrastia R. Gardner R. C. Yaffe K. (2020). Blood biomarkers of traumatic brain injury and cognitive impairment in older veterans. Neurology 95:e1126–e1133. 10.1212/WNL.0000000000010087 32571850 PMC7538225

[B104] Perets N. Hertz S. London M. Offen D. (2018). Intranasal administration of exosomes derived from mesenchymal stem cells ameliorates autistic-like behaviors of BTBR mice. Mol. Autism 9:57. 10.1186/s13229-018-0240-6 30479733 PMC6249852

[B105] Perets N. Oron O. Herman S. Elliott E. Offen D. (2020). Exosomes derived from mesenchymal stem cells improved core symptoms of genetically modified mouse model of autism Shank3B. Mol. Autism 11:65. 10.1186/s13229-020-00366-x 32807217 PMC7433169

[B106] Perez-Gonzalez R. Kim Y. Miller C. Pacheco-Quinto J. Eckman E. A. Levy E. (2020). Extracellular vesicles: where the amyloid precursor protein carboxyl-terminal fragments accumulate and amyloid-beta oligomerizes. FASEB J. 34, 12922–12931. 10.1096/fj.202000823R 32772431 PMC7496786

[B107] Perucca P. Bahlo M. Berkovic S. F. (2020). The Genetics of Epilepsy. Annu. Rev. Genomics Hum. Genet. 21, 205–230. 10.1146/annurev-genom-120219-074937 32339036

[B108] Pieragostino D. Cicalini I. Lanuti P. Ercolino E. Di Ioia M. Zucchelli M.. (2018). Enhanced release of acid sphingomyelinase-enriched exosomes generates a lipidomics signature in CSF of Multiple Sclerosis patients. Sci. Rep. 8:3071. 10.1038/s41598-018-21497-5 29449691 PMC5814401

[B109] Pinnell J. R. Cui M. Tieu K. (2021). Exosomes in Parkinson disease. J. Neurochem. 157, 413–428. 10.1111/jnc.15288 33372290 PMC8863192

[B110] Pinto S. Cunha C. Barbosa M. Vaz A. R. Brites D. (2017). Exosomes from NSC-34 cells transfected with hSOD1-G93A are enriched in miR-124 and drive alterations in microglia phenotype. Front. Neurosci. 11:273. 10.3389/fnins.2017.00273 28567000 PMC5434170

[B111] Podvin S. Jones A. Liu Q. Aulston B. Ransom L. Ames J.. (2020). Dysregulation of exosome cargo by mutant tau expressed in human-induced pluripotent stem cell (iPSC) neurons revealed by proteomics analyses. Mol. Cell. Proteomics. 19, 1017–1034. 10.1074/mcp.RA120.002079 32295833 PMC7261814

[B112] Qi Y. Guo L. Jiang Y. Shi Y. Sui H. Zhao L. (2020). Brain delivery of quercetin-loaded exosomes improved cognitive function in AD mice by inhibiting phosphorylated tau-mediated neurofibrillary tangles. Drug Deliv. 27, 745–755. 10.1080/10717544.2020.1762262 32397764 PMC7269046

[B113] Rahmani A. Saleki K. Javanmehr N. Khodaparast J. Saadat P. Nouri H. R. (2020). Mesenchymal stem cell-derived extracellular vesicle-based therapies protect against coupled degeneration of the central nervous and vascular systems in stroke. Ageing Res. Rev. 62:101106. 10.1016/j.arr.2020.101106 32565329

[B114] Ratajczak M. Z. Ratajczak J. (2020). Extracellular microvesicles/exosomes: discovery, disbelief, acceptance, and the future? Leukemia 34, 3126–3135. 10.1038/s41375-020-01041-z 32929129 PMC7685969

[B115] Reed E. R. Latourelle J. C. Bockholt J. H. Bregu J. Smock J. Paulsen J. S.. (2018). MicroRNAs in CSF as prodromal biomarkers for Huntington disease in the PREDICT-HD study. Neurology 90:e264–e272. 10.1212/WNL.0000000000004844 29282329 PMC5798654

[B116] Riazifar M. Mohammadi M. R. Pone E. J. Yeri A. Lasser C. Segaliny A. I.. (2019). Stem cell-derived exosomes as nanotherapeutics for autoimmune and neurodegenerative disorders. ACS Nano 13, 6670–6688. 10.1021/acsnano.9b01004 31117376 PMC6880946

[B117] Ruan Z. Delpech J. C. Venkatesan Kalavai S. Van Enoo A. A. Hu J. Ikezu S.. (2020). P2RX7 inhibitor suppresses exosome secretion and disease phenotype in P301S tau transgenic mice. Mol. Neurodegener. 15:47. 10.1186/s13024-020-00396-2 32811520 PMC7436984

[B118] Ryskalin L. Busceti C. L. Biagioni F. Limanaqi F. Familiari P. Frati A.. (2019). Prion protein in glioblastoma multiforme. Int. J. Mol. Sci. 20:5107. 10.3390/ijms20205107 31618844 PMC6834196

[B119] Sala M. Hollinger K. R. Thomas A. G. Dash R. P. Tallon C. Veeravalli V.. (2020). Novel human neutral sphingomyelinase 2 inhibitors as potential therapeutics for Alzheimer disease. J. Med. Chem. 63, 6028–6056. 10.1021/acs.jmedchem.0c00278 32298582 PMC8025741

[B120] Silverman J. M. Christy D. Shyu C. C. Moon K. M. Fernando S. Gidden Z.. (2019). CNS-derived extracellular vesicles from superoxide dismutase 1 (SOD1) (G93A) ALS mice originate from astrocytes and neurons and carry misfolded SOD1. J. Biol. Chem. 294, 3744–3759. 10.1074/jbc.RA118.004825 30635404 PMC6416428

[B121] Singh P. K. Muqit M. M. K. (2020). Parkinson's: a disease of aberrant vesicle trafficking. Annu. Rev. Cell Dev. Biol. 36, 237–264. 10.1146/annurev-cellbio-100818-125512 32749865

[B122] Soares Martins T. Marcalo R. Ferreira M. Vaz M. Silva R. M. Martins Rosa I.. (2021a). Exosomal abeta-binding proteins identified by “*in silico*” analysis represent putative blood-derived biomarker candidates for Alzheimer's disease. Int. J. Mol. Sci. 22:3933. 10.3390/ijms22083933 33920336 PMC8070602

[B123] Soares Martins T. Trindade D. Vaz M. Campelo I. Almeida M. Trigo G.. (2021b). Diagnostic and therapeutic potential of exosomes in Alzheimer's disease. J. Neurochem. 156, 162–181. 10.1111/jnc.15112 32618370

[B124] Suk T. R. Rousseaux M. W. C. (2020). The role of TDP-43 mislocalization in amyotrophic lateral sclerosis. Mol. Neurodegener. 15:45. 10.1186/s13024-020-00397-1 32799899 PMC7429473

[B125] Sun T. Ding Z. X. Luo X. Liu Q. S. Cheng Y. (2020). Blood exosomes have neuroprotective effects in a mouse model of Parkinson's disease. Oxid. Med. Cell. Longev. 2020:3807476. 10.1155/2020/3807476 33294121 PMC7714585

[B126] Tan S. K. Pastori C. Penas C. Komotar R. J. Ivan M. E. Wahlestedt C.. (2018). Serum long noncoding RNA HOTAIR as a novel diagnostic and prognostic biomarker in glioblastoma multiforme. Mol. Cancer 17:74. 10.1186/s12943-018-0822-0 29558959 PMC5861620

[B127] Thery C. Witwer K. W. Aikawa E. Alcaraz M. J. Anderson J. D. Andriantsitohaina R.. (2018). Minimal information for studies of extracellular vesicles 2018 (MISEV2018): a position statement of the International Society for Extracellular Vesicles and update of the MISEV2014 guidelines. J. Extracell. Vesicles 7:1535750. 10.1080/20013078.2018.1535750 30637094 PMC6322352

[B128] Tsunemi T. Ishiguro Y. Yoroisaka A. Hattori N. (2021). Analysis of alpha-synuclein in exosomes. Methods Mol. Biol. 2322, 41–45. 10.1007/978-1-0716-1495-24 34043190

[B129] Tsunemi T. Ishiguro Y. Yoroisaka A. Valdez C. Miyamoto K. Ishikawa K.. (2020). Astrocytes protect human dopaminergic neurons from alpha-synuclein accumulation and propagation. J. Neurosci. 40, 8618–8628. 10.1523/JNEUROSCI.0954-20.2020 33046546 PMC7643299

[B130] Ueno Y. Hira K. Miyamoto N. Kijima C. Inaba T. Hattori N. (2020). Pleiotropic effects of exosomes as a therapy for stroke recovery. Int. J. Mol. Sci. 21:6894. 10.3390/ijms21186894 32962207 PMC7555640

[B131] Vaidya M. Sugaya K. (2020). Differential sequences and single nucleotide polymorphism of exosomal SOX2 DNA in cancer. PLoS ONE 15:e0229309. 10.1371/journal.pone.0229309 32092088 PMC7039433

[B132] Vandendriessche C. Bruggeman A. Van Cauwenberghe C. Vandenbroucke R. E. (2020). Extracellular vesicles in Alzheimer's and Parkinson's disease: small entities with large consequences. Cells 9:2485. 10.3390/cells9112485 33203181 PMC7696752

[B133] Venkat P. Cui C. Chopp M. Zacharek A. Wang F. Landschoot-Ward J.. (2019). MiR-126 mediates brain endothelial cell exosome treatment-induced neurorestorative effects after stroke in type 2 diabetes mellitus mice. Stroke. 50, 2865–2874. 10.1161/STROKEAHA.119.025371 31394992 PMC6756941

[B134] Wang B. Han S. (2019). Modified exosomes reduce apoptosis and ameliorate neural deficits induced by traumatic brain injury. ASAIO J. 65, 285–292. 10.1097/MAT.0000000000000810 29762232

[B135] Wang B. Wu Z. H. Lou P. Y. Chai C. Han S. Y. Ning J. F.. (2019a). Human bone marrow-derived mesenchymal stem cell-secreted exosomes overexpressing microRNA-34a ameliorate glioblastoma development via down-regulating MYCN. Cell Oncol. 42, 783–799. 10.1007/s13402-019-00461-z 31332647 PMC12994354

[B136] Wang J. K. T. Langfelder P. Horvath S. Palazzolo M. J. (2017). Exosomes and homeostatic synaptic plasticity are linked to each other and to huntington's, parkinson's, and other neurodegenerative diseases by database-enabled analyses of comprehensively curated datasets. Front. Neurosci. 11:149. 10.3389/fnins.2017.00149 28611571 PMC5374209

[B137] Wang M. M. Feng Y. S. Tan Z. X. Xing Y. Dong F. Zhang F. (2020a). The role of exosomes in stroke. Mol. Biol. Rep. 47, 6217–6228. 10.1007/s11033-020-05569-2 32514999

[B138] Wang Q. Han C. L. Wang K. L. Sui Y. P. Li Z. B. Chen N.. (2019b). Integrated analysis of exosomal lncRNA and mRNA expression profiles reveals the involvement of lnc-MKRN2-42:1 in the pathogenesis of Parkinson's disease. CNS Neurosci. Ther. 26, 527–537. 10.1111/cns.13277 31814304 PMC7163584

[B139] Wang X. Zhou Y. Gao Q. Ping D. Wang Y. Wu W.. (2020b). The role of exosomal microRNAs and oxidative stress in neurodegenerative diseases. Oxid. Med. Cell. Longev. 2020:3232869. 10.1155/2020/3232869 33193999 PMC7641266

[B140] Wang Y. Balaji V. Kaniyappan S. Kruger L. Irsen S. Tepper K.. (2017). The release and trans-synaptic transmission of Tau via exosomes. Mol. Neurodegener. 12:5. 10.1186/s13024-016-0143-y 28086931 PMC5237256

[B141] Wei Z. X. Xie G. J. Mao X. Zou X. P. Liao Y. J. Liu Q. S.. (2020). Exosomes from patients with major depression cause depressive-like behaviors in mice with involvement of miR-139-5p-regulated neurogenesis. Neuropsychopharmacology. 45, 1050–1058. 10.1038/s41386-020-0622-2 31986519 PMC7162931

[B142] Williams A. M. Bhatti U. F. Brown J. F. Biesterveld B. E. Kathawate R. G. Graham N. J.. (2020). Early single-dose treatment with exosomes provides neuroprotection and improves blood-brain barrier integrity in swine model of traumatic brain injury and hemorrhagic shock. J. Trauma Acute Care Surg. 88, 207–218. 10.1097/TA.0000000000002563 31804413

[B143] Xian P. Hei Y. Wang R. Wang T. Yang J. Li J.. (2019). Mesenchymal stem cell-derived exosomes as a nanotherapeutic agent for amelioration of inflammation-induced astrocyte alterations in mice. Theranostics 9, 5956–5975. 10.7150/thno.33872 31534531 PMC6735367

[B144] Xiao Y. Geng F. Wang G. Li X. Zhu J. Zhu W. (2018). Bone marrow-derived mesenchymal stem cells-derived exosomes prevent oligodendrocyte apoptosis through exosomal miR-134 by targeting caspase-8. J. Cell Biochem. 10.1002/jcb.27519. [Epub ahead of print]. 30191592

[B145] Xin H. Katakowski M. Wang F. Qian J. Y. Liu X. S. Ali M. M.. (2017). MicroRNA cluster miR-17-92 cluster in exosomes enhance neuroplasticity and functional recovery after stroke in rats. Stroke 48, 747–753. 10.1161/STROKEAHA.116.015204 28232590 PMC5330787

[B146] Xin H. Liu Z. Buller B. Li Y. Golembieski W. Gan X.. (2021). MiR-17-92 enriched exosomes derived from multipotent mesenchymal stromal cells enhance axon-myelin remodeling and motor electrophysiological recovery after stroke. J. Cereb. Blood Flow Metab. 41, 1131–1144. 10.1177/0271678X20950489 32811262 PMC8054728

[B147] Xing Y. Bai Y. (2020). A review of exercise-induced neuroplasticity in ischemic stroke: pathology and mechanisms. Mol. Neurobiol. 57, 4218–4231. 10.1007/s12035-020-02021-1 32691303

[B148] Xu X. Zhuang C. Chen L. (2020). Exosomal long non-coding RNA Expression from serum of patients with acute minor stroke. Neuropsychiatr. Dis. Treat. 16, 153–160. 10.2147/NDT.S230332 32021207 PMC6968802

[B149] Yang J. Luo S. Zhang J. Yu T. Fu Z. Zheng Y.. (2021). Exosome-mediated delivery of antisense oligonucleotides targeting alpha-synuclein ameliorates the pathology in a mouse model of Parkinson's disease. Neurobiol. Dis. 148:105218. 10.1016/j.nbd.2020.105218 33296726

[B150] Yang Y. Cai Y. Zhang Y. Liu J. Xu Z. (2018). Exosomes secreted by adipose-derived stem cells contribute to angiogenesis of brain microvascular endothelial cells following oxygen-glucose deprivation *in vitro* through MicroRNA-181b/TRPM7 axis. J. Mol. Neurosci. 65, 74–83. 10.1007/s12031-018-1071-9 29705934

[B151] Yang Z. Wang T. Wu D. Min Z. Tan J. Yu B. (2020). RNA N6-methyladenosine reader IGF2BP3 regulates cell cycle and angiogenesis in colon cancer. J Exp Clin Cancer Res. 39:203. 10.1186/s13046-020-01714-8 32993738 PMC7523351

[B152] Yin Z. Han Z. Hu T. Zhang S. Ge X. Huang S.. (2020). Neuron-derived exosomes with high miR-21-5p expression promoted polarization of M1 microglia in culture. Brain Behav. Immun. 83, 270–282. 10.1016/j.bbi.2019.11.004 31707083

[B153] Yue X. Lan F. Xia T. (2019). Hypoxic glioma cell-secreted exosomal miR-301a activates wnt/beta-catenin signaling and promotes radiation resistance by targeting TCEAL7. Mol Ther. 27, 1939–1949. 10.1016/j.ymthe.2019.07.011 31402274 PMC6838947

[B154] Zeng A. Wei Z. Yan W. Yin J. Huang X. Zhou X.. (2018). Exosomal transfer of miR-151a enhances chemosensitivity to temozolomide in drug-resistant glioblastoma. Cancer Lett. 436, 10–21. 10.1016/j.canlet.2018.08.004 30102952

[B155] Zhang Y. Bi J. Huang J. Tang Y. Du S. Li P. (2020). Exosome: a review of its classification, isolation techniques, storage, diagnostic and targeted therapy applications. Int. J. Nanomed. 15, 6917–6934. 10.2147/IJN.S264498 33061359 PMC7519827

[B156] Zhang Y. Zhang Y. Chopp M. Pang H. Zhang Z. G. Mahmood A.. (2021). MiR-17-92 cluster-enriched exosomes derived from human bone marrow mesenchymal stromal cells improve tissue and functional recovery in rats after traumatic brain injury. J. Neurotrauma 38, 1535–1550. 10.1089/neu.2020.7575 33787364 PMC8126425

[B157] Zhao C. Deng Y. He Y. Huang X. Wang C. Li W. (2021). Decreased level of exosomal mir-5121 released from microglia suppresses neurite outgrowth and synapse recovery of neurons following traumatic brain injury. Neurotherapeutics. 10.1007/s13311-020-00999-z. [Epub ahead of print]. 33475953 PMC8423926

[B158] Zhao J. Liu X. Xia W. Zhang Y. Wang C. (2020). Targeting amyloidogenic processing of APP in Alzheimer's disease. Front. Mol. Neurosci. 13:137. 10.3389/fnmol.2020.00137 32848600 PMC7418514

[B159] Zou J. Guo Y. Wei L. Yu F. Yu B. Xu A. (2020). Long noncoding RNA POU3F3 and alpha-synuclein in plasma L1CAM exosomes combined with beta-glucocerebrosidase activity: potential predictors of Parkinson's disease. Neurotherapeutics. 17, 1104–1119. 10.1007/s13311-020-00842-5 32236821 PMC7609611

